# Supplementary material for: Identification of photoperiod-induced specific miRNAs in the adrenal glands of Sunite sheep (Ovis aries)
Source: Front Vet Sci. 2022 Jul 22;9:888207. doi: 10.3389/fvets.2022.888207 (PMC9354845; doi:10.3389/fvets.2022.888207)
Supplement: Supplementary file 1 [file Data_Sheet_1.ZIP › Supplementary file/miRDeep2 file.zip!/miRDeep2 file/Know_Structure/LP42A2/oar-mir-431.pdf]

miRBase precursor	: oar-mir-431
Total read count	: 159
oar-miR-431 read count	: 41
remaining reads	: 118

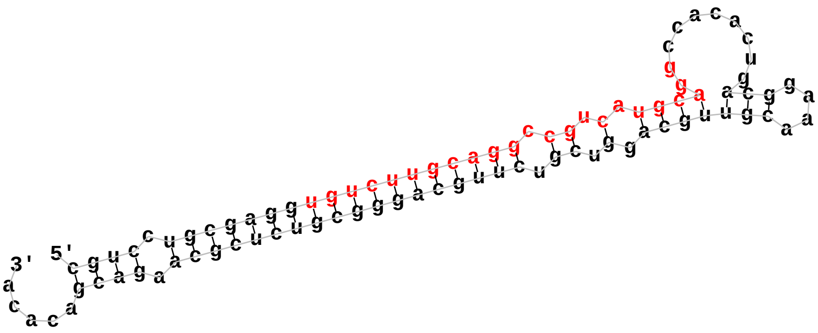

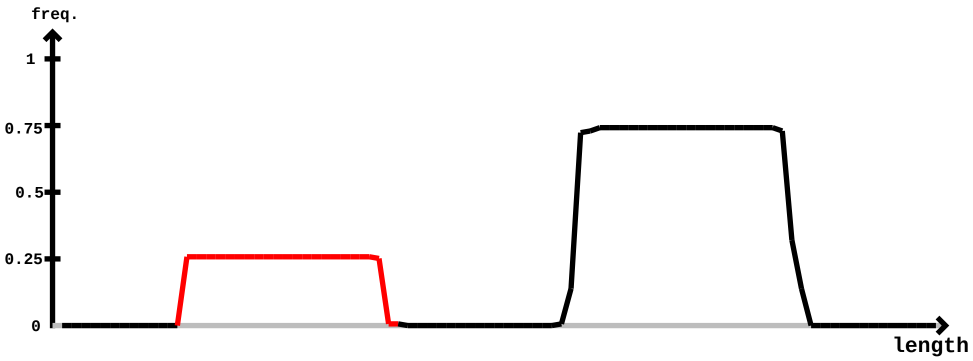


```
               oar-miR-431
5'- cguccugcgaggugucuugcaggccgucaugcaggccacacugacggaaacguugcaggucgucuugcagggcgucucgcaagacgacaca   -3'    exp
    ((((.((((((((((((((((((.((.(.((((..........(((....))))))).).)).))))))))))))))))))).)))).....    reads      mm         sample
    ............ugucuugcaggccgucaugc...........................................................    1          0          seq
    ............ugucuugcaggccgucaugca..........................................................    39         0          seq
    ............ugucuugcaggccgucaugcagg........................................................    1          0          seq
    ....................................................guugcaggucgucuugcagggcguc..............    1          0          seq
    .....................................................uugcaggucgucuugcagggcgu...............    13         0          seq
    .....................................................uugcaggucgucuugcagggcguc..............    7          0          seq
    .....................................................uugcaggucgucuugcagggcgucu.............    1          0          seq
    ......................................................ugcaggucgucuugcagggcg................    2          0          seq
    ......................................................ugcaggucgucuugcagggcgu...............    50         0          seq
    ......................................................ugcaggucgucuugcagggcguc..............    20         0          seq
    ......................................................ugcaggucgucuugcagggcgucu.............    21         0          seq
    .......................................................gcaggucgucuugcagggcguc..............    1          0          seq
    ........................................................caggucgucuugcagggcgu...............    2          0          seq
```
